# Active Case Finding for Tuberculosis through TOUCH Agents in Selected High TB Burden Wards of Kolkata, India: A Mixed Methods Study on Outcomes and Implementation Challenges

**DOI:** 10.3390/tropicalmed4040134

**Published:** 2019-11-01

**Authors:** Abhijit Dey, Pruthu Thekkur, Ayan Ghosh, Tanusree Dasgupta, Soumyajyoti Bandopadhyay, Arista Lahiri, Chidananda Sanju S V, Milan K. Dinda, Vivek Sharma, Namita Dimari, Dibyendu Chatterjee, Isita Roy, Anuradha Choudhury, Parthiban Shanmugam, Brojo Kishore Saha, Sanghamitra Ghosh, Sharath Burugina Nagaraja

**Affiliations:** 1 Tuberculosis Health Action Learning Initiative (THALI), Kolkata, West Bengal 700107, India; tanusree@cinindia.org (T.D.); milan@whpindia.org (M.K.D.); vivek_sharma@in.jsi.com (V.S.); namita_dimari@in.jsi.com (N.D.); chatterjeephotography@rediffmail.com (D.C.); isita@cinindia.org (I.R.); anuradha@cinindia.org (A.C.); drpartthiban@rediffmail.com (P.S.); 2 International Union against Tuberculosis and Lung Diseases, 75006 Paris, France; Pruthu.TK@theunion.org; 3 The Union South East Asia Office, New Delhi 110016, India; 4 Department of Community Medicine, College of Medicine & JNM Hospital, Kalyani, West Bengal 741235, India; dr.ayanghosh@rediffmail.com (A.G.); drsoumyajyoti@rediffmail.com (S.B.); 5 Department of Community Medicine, College of Medicine & Sagore Dutta Hospital, Kamarhati, Kolkata 700058, India; Arista_rgkar2008@rediffmail.com; 6 District Tuberculosis Officer, Government of Karnataka, Udupi 576104, India; sanjuchida@rediffmail.com; 7 State Tuberculosis Officer, Govt of West Bengal, West Bengal 700091, India; stowb@rntcp.org; 8 General Secretary, Indian Public Health Association (IPHA), HQ, Kolkata 700073, India; dr.sanghamitraghosh@rediffmail.com; 9 Department of Community Medicine, ESIC Medical College and PGIMSR, Bengaluru 560010, India; dr.sharath.n@esic.nic.in

**Keywords:** active case findings, Tuberculosis, TOUCH Agent, high TB burden area, TB surveillance, 4S Screening, THALI Project, SORT IT, operational research

## Abstract

Active case finding (ACF) for tuberculosis (TB) was implemented in 60 selected high TB burden wards of Kolkata, India. Community volunteers called TOUCH (Targeted Outreach for Upliftment of Community Health) agents (TAs) identified and referred presumptive TB patients (PTBPs) to health facilities for TB diagnosis and treatment. We aimed to describe the “care cascade” of PTBPs that were identified during July to December 2018 and to explore the reasons for attrition as perceived by TAs and PTBPs. An explanatory mixed-methods study with a quantitative phase of cohort study using routinely collected data followed by descriptive qualitative study with in-depth interviews was conducted. Of the 3,86242 individuals that were enumerated, 1132 (0.3%) PTBPs were identified. Only 713 (63.0%) PTBPs visited a referred facility for TB diagnosis. TB was diagnosed in 177 (24.8%). The number needed to screen for one TB patient was 2183 individuals. The potential reasons for low yield were stigma and apprehension about TB, distrust about TA, wage losses for attending health facilities, and substance abuse among PTBPs. The yield of ACF was suboptimal with low PTBP identification rate and a high attrition rate. Interviewing each individual for symptoms of TB and supporting PTBPs for diagnosis through sputum collection and transport can be adopted to improve the yield.

## 1. Introduction

Tuberculosis (TB) remains a major public health problem, with an estimated 10 million TB patients and 1.6 million deaths globally due to TB in the year 2017 [1]. In the same year, only 6.7 million TB patients were referred to national TB programs (NTP) [1]. The remaining 3.3 million (33% of the estimated) were either undetected or were detected but were not referred to NTP. India alone has an estimated 0.8 million patients who were not referred to NTP [1].

TB patients who go undetected or are not treated can be a potential threat to TB control efforts as they contribute to uninterrupted transmission of the disease. One of the potential reasons for undetected TB patients is over reliance on passive case finding for diagnosis of TB in low- and middle-income countries (LMICs) [2,3]. 

The World Health Organization’s (WHO) End TB strategy proposed the early diagnosis of TB through Active case finding (ACF) in high risk groups as a key component to end the TB epidemic by 2030 [4]. ACF requires healthcare providers to actively reach and provide access to TB diagnostic services for communities or population groups that are underserved or at higher risk of TB. 

Systematic reviews have reported ACF to be beneficial in increasing case detection [5,6]. Also, studies have reported ACF to be beneficial in reducing the delay in TB diagnosis, out of pocket expenditure, unsuccessful treatment outcomes, and incidence of TB [3,7,8]. The benefits of community based ACF activity depends on the selection of high risk groups, prevalence of TB in the selected group, incentives provided to healthcare workers, the diagnostic algorithm adopted, and support provided to patients in completing the cascade of TB diagnosis and treatment. 

The ACF activity has sequential stages (“cascade of care”) through which the patient traverses from diagnosis to treatment. Nevertheless, studies elsewhere have shown as high as 46% attrition rates at various stages of diagnosis and treatment cascade [9,10]. Thus, it is essential for ACF projects to monitor attrition at different stages, the yield of TB patients, reasons for attrition, and challenges in implementing ACF activity. This information can help programme managers to make informed decisions to optimize ACF activity services and the benefits of ACF.

The National Strategic Plan for TB elimination [2017–25] of India recommended ACF among high risk groups as a strategy to detect the “missing” TB patients [11]. Since mid-2018, the NTP conducted five rounds of two week long ACF activity among identified marginalized and vulnerable groups in selected districts of the country. The NTP also encouraged partner agencies supporting TB control to implement ACF among high risk groups [11]. 

The United States Agency for International Development (USAID) funded Tuberculosis Health Action Learning Initiative (THALI) project in West Bengal implemented ACF activity in 60 high TB burden wards of Kolkata Municipal Corporation (KMC) [12,13,14,15,16]. A part-time, salaried community health volunteer known as a Targeted Outreach for Upliftment of Community Health (TOUCH) agent was deployed in each ward to engage with the community and conduct ACF. The THALI project ACF was different from that conducted by NTP as the activity was conducted throughout the year by a TOUCH agent (TA) who is not part of the general health system. 

There is limited information on the process, yield, and challenges in implementing ACF activities in India. Although the THALI project has been implementing ACF since 2018, there has been no systematic evaluation of the programme. The information and lessons learnt from the programme is imperative to fix the deficiencies and improve the efficiency of ACF conducted by THALI and also NTP.

Hence, we aimed to determine the proportion identified as presumptive TB patient (PTBP), reaching the health facility for diagnosis, diagnosed with TB, and TB treatment initiated among individuals enumerated by TAs during THALI project ACF activity conducted between July and December 2018. Also, we aimed to explore the challenges in implementing the ACF activity as perceived by TAs and PTBPs.

## 2. Materials and Methods 

### 2.1. Study Design

We conducted an explanatory mixed methods study with the quantitative part (cohort study using secondary data collected routinely by the THALI Project) followed by a qualitative part (descriptive study) [17].

### 2.2. Study Setting

Kolkata is a capital of West Bengal, India. It is one of the largest metro cities in the country with a population of 4.5 million [18]. The Kolkata Municipal Corporation (KMC) is divided into 144 urban wards as administrative units. The KMC has 5600 recognized slums and about 1.4 million individuals (~33% of the total population) reside in slums [19,20].

The KMC is divided into 10 urban health districts and each district has a TB officer who reports to the city TB officer, a nodal person for the delivery of NTP services in KMC. In total, there are 21 tuberculosis units (TU) and 42 designated microscopy centers (DMCs) in KMC [20]. The USAID supported THALI project has implemented ACF activity in 60 high TB burden wards of KMC since June 2018 through TAs. 

#### 2.2.1. Ward Selection

**Figure 1:**
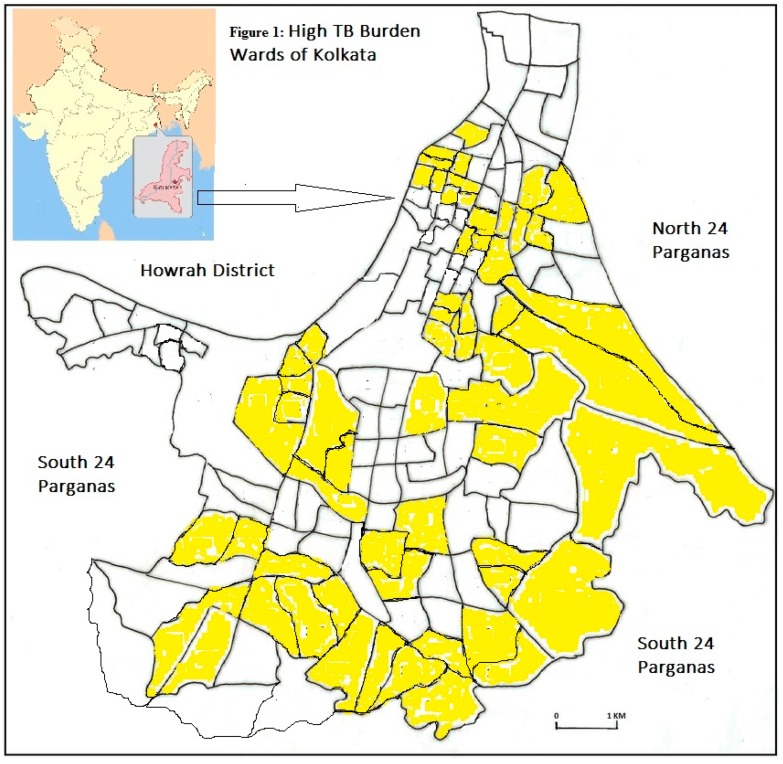
High Tuberculosis (TB) Burden Wards (yellow) of Kolkata Municipal Corporation (KMC).

The wards for ACF were identified based on the burden of TB cases and the percentage of slum population. The number of TB patients notified in each of the 144 wards during 1st March–31st May 2018 was calculated. The 54 wards with 10 or more cases were considered as high TB burden wards. Six other wards with a high proportion of slum population were considered and in total, 60 wards were identified as high priority wards for ACF activity (Figure 1).

#### 2.2.2. TOUCH Agent (TA) 

The TAs are mainly female community volunteers residing in the same ward with the ability to read and write the English language. The selected TAs are trained by medical officers of the THALI project. Two days of induction training on the basics of TB, ACF for TB, NTP diagnostic algorithm, and patient support during TB treatment are provided. The TA spends at least 3 h per day, six days a week in the ward conducting ACF and community sensitization on TB. During each day, the TA visits 20 houses for ACF. The TA is paid a fixed compensation of 3000 INR (~ 43 USD) per month.

#### 2.2.3. Active Case Finding 

During house visits, the TA asks the available individuals about the presence of symptoms that are indicative of TB (persistent cough for ≥ 2 weeks, fever for ≥ 2 weeks, significant weight loss (>5% weight loss over the last 3 months), blood in sputum, and night sweats ≥ 2 weeks). The TA also enquires about the presence of symptoms that are indicative of TB among those who are not available at the home by speaking to the adult informant. 

Those with symptoms that are suggestive of TB are considered as PTBP and are referred to either the nearest DMC or the private practitioner based on the patient’s preference. All the PTBPs referred are provided with referral slips that are signed by the TA. At the health facilities, PTBPs are evaluated for TB using sputum smear microscopy, chest X-ray, and Xpert MTB/Rif assay as per the NTP diagnostic algorithm [21].

**Figure 2:**
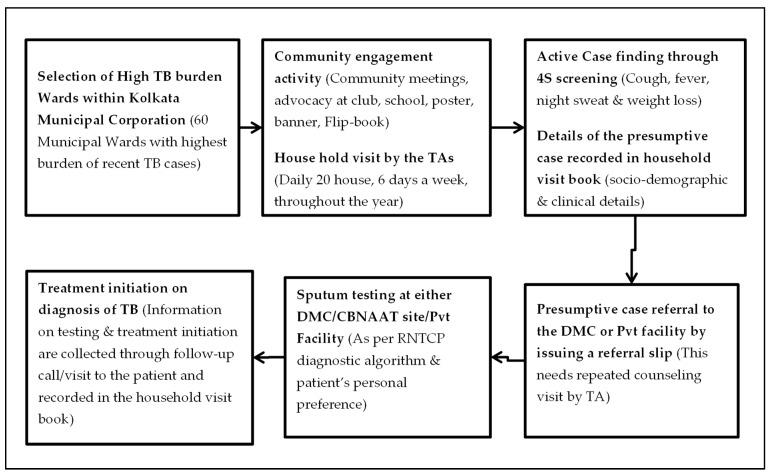
Flow Diagram of the ACF activity by the TAs under the THALI Project. **Abbreviations:** TA = Touch Agent; DMC = Designated Microscopy Center; CBNAAT = Cartridge Based Nucleic Acid Amplification Test; RNTCP = Revised National Tuberculosis Control Program.

The TA re-visits the house of the PTBP within seven days to collect the information about their visit to the referred health facility. In cases when the patient has failed to visit the health facility, the TA counsels the patient and a repeat house visit is made. If the patient has made the visit, the results of the diagnostic test and details of the treatment initiation are collected. Also, those diagnosed with TB are visited at least three times for contact tracing and counselling support during TB treatment (Figure 2).

#### 2.2.4. Recording 

The TA maintains a “Household visit book” and documents the number of individuals screened and the number of individuals with chest symptoms. The socio-demographic, clinical, referral, and treatment details of the PTBP are noted in the “Household visit book”. The field coordinator reviews the “Household visit book” for completeness during supervision visits. 

### 2.3. Study Population

#### 2.3.1. Quantitative 

We included all the PTBPs identified by the TAs between July and December 2018 during the ACF activity conducted in the selected wards of KMC. 

#### 2.3.2. Qualitative 

We included TAs (n = 10) and PTBPs (n = 7) who were identified during the study reference period. TA’s were purposively selected using maximum variation sampling with the selection of those who had referred a maximum and minimum number of PTBPs. The PTBPs were purposively selected using the extreme variation sampling based on whether he/she attended the health facility on referral. The final sample size was guided by the saturation of findings.

### 2.4. Data Variables, Sources of Data, and Data Collection

#### 2.4.1. Quantitative 

Using structured proforma, a principal investigator extracted the details of PTBPs from the “household visit book” during February 2019. The date of house visit, ward number, age, gender, history of previous TB, family history of TB, tobacco use, alcohol use, diabetes, HIV, presenting symptoms, referral site, visited referred facility, date of visit to the referred facility, presence of pulmonary TB, date of TB diagnosis, status of treatment initiation, and date of treatment initiation were extracted. 

#### 2.4.2. Qualitative 

In-depth interviews were conducted face-to-face in local language (Bengali) by AD (a male medical doctor, MPH, trained in qualitative research) and were audio recorded using a mobile voice recorder after obtaining consent. The interviewer was working in a THALI project, but was not involved in the implementation of the ACF activity. Separate interview schedules were used to interview TAs and PTBPs. The TA’s were interviewed at their residence or workplace. PTBPs were interviewed at their residence. Interviews lasted for an average of 21 min (range 8–42 min). At the end of each interview, debriefing was done to ensure member checking.

### 2.5. Data Entry and Analysis

#### 2.5.1. Quantitative 

Data was double-entered and validated using EpiData entry version 3.1 (EpiData Association, Odense, Denmark). The analysis was done using Stata 11.0 (StataCorp LP, College Station, TX, USA). Key analytic outputs were the number and proportion of PTBPs at each step of the diagnosis–treatment pathway. The percentage with 95% CI was used to summarize the proportion of PTBPs at each step. 

To assess the independent association of sociodemographic and clinical characteristics of PTBPs with “not visiting referred health facility for diagnosis”, a multivariable model (modified Poisson regression) with cluster adjustment at the ward level was used to calculate adjusted relative risk (aRR) with 95% confidence intervals (CI). Similarly, a multivariable model (modified Poisson regression) with cluster adjustment at the ward level was used to assess the factors that were independently associated with “diagnosis of TB” among those who reached the health facility after referral. A p value of < 0.05 was considered to be statistically significant.

#### 2.5.2. Qualitative 

Transcripts were prepared on the same day or one day after the interview using audio-recording and field notes. Manual descriptive thematic analysis of the transcripts was done by the AD and PT to identify the codes. The decision on the final coding and theme generation was done using standard procedures and was in consensus. The findings were reported using “Consolidated Criteria for Reporting Qualitative Research” [COREQ guideline] [22].

### 2.6. Ethical Approval

The study was approved by the Institutional Ethics Committee of the College of Medicine & JNM Hospital, Kalyani, West Bengal (F-24/PR/COMJNMH/IEC/19/1428) and the Ethics Advisory Group of the International Union against Tuberculosis and Lung Disease, Paris, France (EAG-130/18). The committees provided a waiver for obtaining written consent from PTBPs as we used routinely collected secondary data. Written informed consent for conducting an in-depth interview and audio-recording the interviews was obtained from all the participants who were included in the qualitative study.

## 3. Results

### 3.1. Quantitative

**Figure 3:**
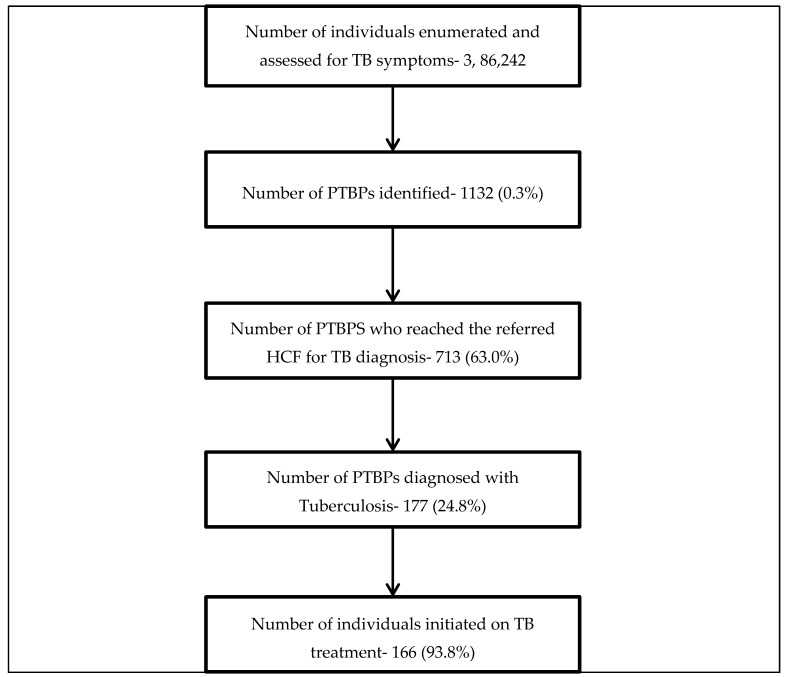
Flowchart depicting the number and percentage of participants at different stages of the diagnostic and treatment cascade test among PTBPs identified by ACF activity in Kolkata West Bengal India during July–December 2018. **Abbreviations:** ACF = Active Case Findings; PTBP = Presumptive TB Patient; HCF = Health Care Facility.

Of the total 386,242 individuals who were enumerated by TAs from 92,294 houses, 1132 (0.3%) were identified as PTBPs (Figure 3). 

The mean age of the PTBPs was 43.1 (standard deviation: 17.1) years and 647 (57.2%) were males. Tobacco use in the last month was reported by 175 (15.5%) PTBPs and 45 (3.9%) reported the use of alcohol in the past month. Known diabetes was reported by 244 (21.5%) and 13 (1.2%) reported to have HIV infection. Of the total, 84 (7.4%) had previous history of TB and 118 (10.4%) gave history of TB among family members in the last two years. A cough of ≥ 2 weeks without any other symptoms that were indicative of TB was seen in 687 (60.7%) (Table 1).

Of the 1132 PTBPs, 713 (63.0%, 95% CI: 60.1%–65.8%) visited the referred health facility for diagnosis of TB. Among those who visited a health facility, the median duration between referral and reaching the facility was 2 (inter-quartile range 1–6) days. Table 2 shows the factors associated with not visiting the referred health facility among PTBPs. Female gender (aRR-1.3 (95% CI: 1.1–1.5)), alcohol use (aRR-1.7 (95% CI: 1.3–2.2)), cough as the only symptom (aRR-1.8 (95% CI: 1.5–2.2)), and family history of TB (aRR-1.3 (95% CI: 1.1–1.5)) were independently associated with not visiting the referred health facility.

Of the 713 PTBPs visiting the health facility for diagnosis, 177 (24.8%) were diagnosed with TB. Being aged less than 30 years (aRR-2.4 (95% CI: 1.6–3.5)), the presence of a cough along with other symptoms (aRR-3.3 (95% CI: 2.4–4.4)), no previous history of TB (aRR-3.6 (95% CI: 1.7–7.6)), and family history of TB (aRR-2.7 (95% CI: 2.0–3.6) were independently associated with the presence of TB (Table 3). The number needed to screen (NNS) one TB patient among all those enumerated during the ACF activity was 2183 (386242/177) individuals. 

### 3.2. Qualitative

**Figure 4:**
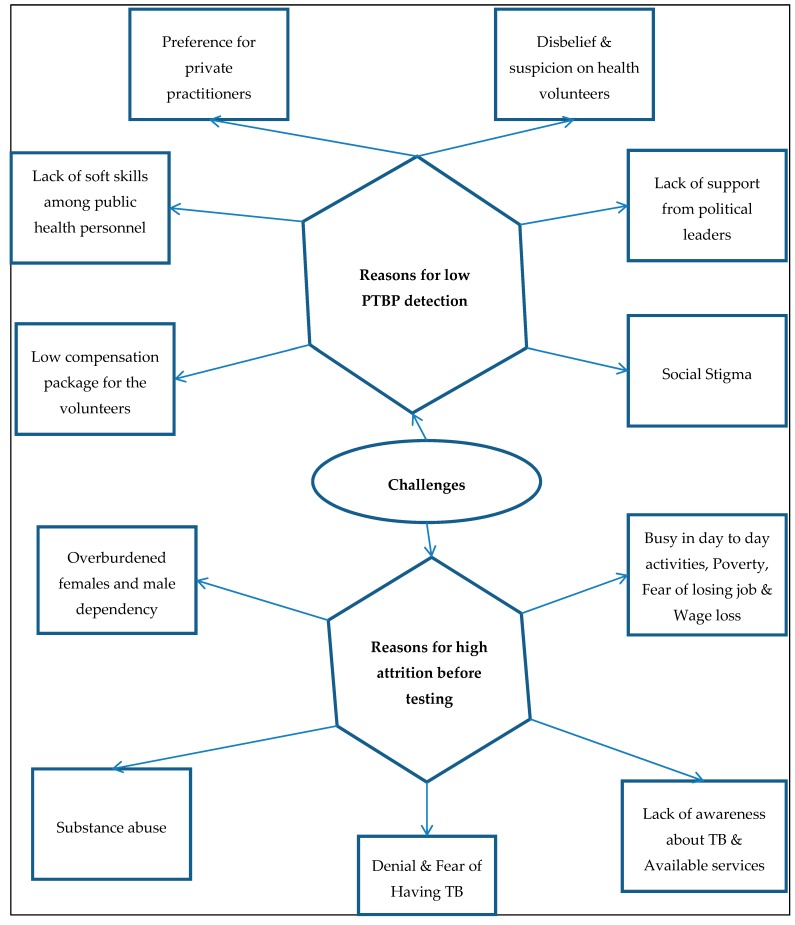
Themes and categories related to the challenges in the implementation of ACF activity in high TB burden wards of Kolkata, 2018.

We deduced 38 codes, which were clubbed into 11 categories. The categories are described below under two broad themes, (1) Challenges in house to house visits and identification of PTBP and (2) Challenges in visiting the referred health facility for TB diagnosis (Figure 4).

#### 3.2.1. Challenges in House to House Visits and Identification of PTBP: 

The codes that were related to this theme were grouped and summarized into six categories. 

(1) Social stigma

The TAs reported that there is social stigma around TB which is found to be a deterrent for the activity. They felt that people do not disclose the presence of PTBP in their house if neighbors are around during the interview.

“When first time we visit any community or household and asked about whether anybody having symptoms like TB, usually none responds on spot. But after coming back, they call us that there is a TB suspect in their family. At the time of my visit, there were many people so they didn’t tell that time.”—38 year old female TA.

(2) Lack of support from political leaders 

The TAs reported the interference of local political leaders in their activity. Political leaders deny the presence of disease in their wards and ask the TAs not to conduct ACF activity. 

“They ask ‘why you people come to my house often? I don’t have time to entertain your queries. There is no TB in my Ward’, despite having lots TB patients in and around the party office!”—27 years old female TA.

(3) Suspicion about TAs

The TAs felt that people were suspicious about them. The people were not aware of the ACF activity and assumed that the TAs visited the houses with some hidden agenda.

“The community dwellers and the local club initially did not believe that there are many TB patients in their area and house to house visit is needed to detect more TB case. They were thinking that we have some other motive!”—55 years old female TA.

(4) Preference for private practitioners 

The TAs felt that the community has a preference for private healthcare providers. Thus, they do not want to disclose the details of their symptoms with TAs as they consider them to be from the public health system.

“For any illness, here, people prefer to go to Private clinic if they can afford. If can’t spent money, then prefer to go to homeopath, unani or even quacks. For maintaining privacy, also sometime they prefer private clinic. They don’t tell us anything.”—28 years old female TA.

(5) Lack of soft skills among public health personnel

The TAs felt that inappropriate ways of handling patients at the public health facilities further dampen their relationship with the community. They refer the patients to health facilities and patients are treated badly.

“This is actually the biggest challenges for us to get the report timely. When parents visit TU for CBNAAT report, the person sitting at TU sometime behaves rudely and sends back the parents. Sometime they say this test cost 1 lakh so you have to wait! —38 years old female TA.

(6) Unjustified pay for the job done 

The TAs complained that they are paid less than the amount of effort they put into the field. They also saw low salary as one of the reasons for high attrition among the TAs.

“What can we do with this 3000? People in other project with similar job profile getting three four times the salary we are getting. That’s why many people are leaving this project… I’ve also applied for.”—45 years old female TA.

#### 3.2.2. Challenges in PTBPs Visits to Referred Health Facility for TB Diagnosis

The codes related to this theme were grouped and summarized into five categories.

(1) Loss of wages and fear of losing jobs 

The TAs felt that the loss of wages for missing a work-day among daily wage labors and non-availability of leave for salaried employees hinders the PTBPs from visiting the referred health facility. 

“…… she (a PTBP who had not gone for testing) said that she couldn’t go DMC due to fear of wage loss. As sudden leave can trigger deduction of pay and employer may become angry! So, she planned for testing on any suitable next day.”—60 years old female TA.

(2) Lack of awareness about the available TB services at health facilities 

The TAs reported that a few of the PTBPs they had identified had difficulties in identifying a health facility in their locality to access TB services.

“Till now, people are not getting government services in my area, that is ward no. XX, they are not aware of the available government services.”—45 years old female TA.

(3) Denial and apprehension of having the TB disease 

The TAs reported that those PTBPs who have awareness about TB are apprehensive to get themselves tested for the disease. Such patients try to deny that they may have the disease and avoid going to a health facility. 

“I was unable to arrange for sputum test for all identified presumptive case. Because I couldn’t convince few patients that sputum test is needed for them. Patients actually don’t want to believe that they may have TB.”—53 years old female TA.

(4) Substance abuse 

The TAs reported substance abuse among PTBPs as one of the potential reasons for them not visiting the health facilities. They felt it was difficult to get the investigation done for drug addicts and alcoholics.

“Not all patients have gone for testing. Especially who are addicted with hashish (Marijuana) have not gone to DMC, not even given the phone number. I have at least three such case..”—45 years old female TA.

(5) Overburdened females and male dependency 

The TAs felt that it is difficult to mobilize female patients to go to health facilities as they are pre-occupied with household work during the day time. Also, they are dependent on males to take them to health facilities. 

“I have told many times to my husband but he said that he will arrange but he is yet to arrange. I don’t know where to go so I did not go alone.”—29 years old PTBP.

## 4. Discussion

This is one of the first mixed methods studies to assess the “care cascade” and to explore the challenges associated with the implementation of ACF for TB in India. Our study has a few key findings. First, the ACF activity had a low PTBP detection rate with only three out of 1000 apparently healthy individuals enumerated during house-to-house visits having symptoms that were suggestive of TB. Second, there was high attrition of PTBP prior to diagnosis with only two out of three PTBP who were referred actually visiting the health facility. Third, among PTBPs who undergo the diagnostic test, the sputum positivity rate was high, with one out of four having been diagnosed with TB. Fourth, the NNS for one TB patient was relatively high. Fifth, the stigma about TB, suspicion about TA, lack of support from political leaders, and low salary for TAs were the major challenges in conducting house-to-house visit for identifying the PTBPs. Sixth, wage loss, apprehension about the diagnosis, substance abuse, and male dependency of females for seeking care at health facilities were the major challenges for PTBPs to visit the referred health facility. 

We further discuss our study findings in detail. First, the study had a low rate of PTBP detection, whereas the previous studies on ACF have consistently shown high rates of PTBP detection [23,24,25]. The TAs failed to individually enquire about symptoms that are suggestive of TB from all those who were enumerated. As the ACF activity was conducted during the daytime, the symptoms of individuals who were away from home were enquired about by speaking to adult informants. Studies in the past have shown that the detection rate of PTBP may be low if the symptoms are not elicited individually [26]. The informants might not be aware of the TB symptoms and thus, could have failed to identify the PTBPs among those who were absent during the interview. As explored in the qualitative study, the other potential reasons for low rate of PTBP detection are hesitancy to disclose the details of TB symptoms due to stigma and distrust about TAs. Some other studies also revealed similar findings [27,28]. 

The second finding is the high attrition rate among PTBPs referred to health facilities for TB diagnosis. Previous studies have also reported similar findings with attrition rates ranging from 39% to 80% [9,10,29,30]. The rate was high in spite of repeated house visits by TAs to counsel and encourage PTBPs to visit the health facility. The qualitative interviews provided good insight about potential reasons for such high attrition. The wage or work loss due to travel and visits to health facilities during the day time was one of the major reasons. Among females, the attrition rate was high as they were dependent on male family members to visit the health facility. Difficulties in counselling PTBPs with alcohol and other substance abuse also contributed to the attrition. The other important issue was general distrust and unhappiness about the public health system among the public. Thus, these reasons warrant the provision of financial support and assistance to PTBPs to undergo diagnostic tests. 

Third, the rate of TB diagnosis among those PTBPs visiting a health facility was high. This could be due to the fact that only those with perceived need reached the health facility. Though PTBPs were identified by ACF activity, the decision to visit largely depended on the severity of symptoms and thus mimicked the passive case finding. Also, the TAs might have given more preference to referral and linkage of PTBPs with severe and multiple symptoms rather than those with mild symptoms.

Fourth, the overall yield of ACF was low and NNS for TB was high. Previous studies have reported lower NNS on mass screening in high burden TB settings. A potential reason for high NNS might be the low detection and high attrition rate of PTBPs. Although the NNS is high, the activity is still worthwhile as it indirectly sensitizes households on TB symptoms and thus creates awareness in the community. Regular visits to the same community (six days a week) is gradually leading to good rapport building with the community [16]. This might improve the overall case detection in the community and contribute to TB control efforts. 

The study has several strengths. First, it was conducted under programmatic settings and reflects the field realities. Second, we used a mixed methods design, which provided considerable insights into challenges involved in the implementation of ACF and enabled us to interpret the quantitative results. Third, the study has a relatively large sample size and there was no selection bias as we included all the individuals who were enumerated during the study reference period in 60 wards where ACF is implemented. Fourth, we used double data entry and validation using EpiData software and thus data entry errors were minimized. Fifth, we adhered to STrengthening the Reporting of Observational studies in Epidemiology (STROBE) and COREQ guidelines for reporting the study’s findings.

The study has a few limitations. First, as details of diagnostic tests that PTBPs underwent were not recorded routinely, we failed to determine the uptake of various diagnostic tests for TB. Second, as it was a retrospective study using routinely collected data, we failed to capture and adjust our model for potential confounders like socioeconomic status, distance from referred health facility, employment status, education level, and marital status. Thus, the factors associated with “not visiting health facility” among PTBPs have to be interpreted with caution. Third, due to the long duration of anti-TB treatment, we failed to describe the treatment outcomes of TB patients diagnosed through ACF activity. Fourth, the qualitative exploration was limited only to TAs and PTBPs. Thus, we failed to capture experiences of other stakeholders involved in ACF activity like field supervisors, project leads, and programme managers of NTP.

Based on the study’s findings, there are a few implications and recommendations for ACF activities. This model can be replicated in other high TB burden areas considering the following recommendations.

First, house visits need to be scheduled during non-working hours of the day to ensure the presence of the majority of the household members during visits. The feasibility of visits by healthcare workers during early morning or late evening hours needs to be explored. Also, revisits to houses on holidays can be made to probe for symptoms among those who were not present during the initial visit.

Second, the sputum collection and transportation mechanisms need to be established. The healthcare workers can collect the sputum samples from the PTBPs and transport them to diagnostic facilities. Also, the evening clinics can be set up to provide chest x-ray services to working PTBPs. The mobile voice call or SMS reminders to PTBPs can be trialed to improve the uptake of diagnostic tests.

Third, there is need for establishing a robust database management system for tracking of individual PTBPs and programme monitoring. The healthcare workers can enter the PTBPs details directly in the hand held data capture devices instead of documenting it in registers. This will reduce the efforts in digitalizing these registers and data loss. Provision of internet connectivity with the device can be made for real time data sharing. The database also needs to include details on investigations to assess the yield with a combination of various diagnostic tests. The indicators like proportion of PTBPs detected and proportion of PTBPs undergoing diagnostic tests for TB can be included in the programme monitoring. The healthcare workers can be incentivized based on these indicators. 

## 5. Conclusions

The yield of ACF was suboptimal with low PTBP identification rate and high attrition rate. Interviewing each and every individual of the households for symptoms of TB and supporting PTBPs for diagnosis through sputum collection and a transport system can be adopted to improve the yield.

## Figures and Tables

**Table 1:** Sociodemographic and clinical profile of the PTBPs identified by ACF activity in Kolkata Municipal Corporation, West Bengal, India during July–Decemeber 2018, N = 1132.

Characteristics	n	(%)
**Age in Years**		
0–14	36	(3.2)
15–29	206	(18.2)
30–44	323	(28.5)
45–59	280	(24.7)
60–74	184	(16.3)
75 and above	32	(2.8)
Not recorded	71	(6.3)
**Gender**		
Male	647	(57.2)
Female	485	(42.8)
Alcohol use *		
Yes	45	(3.9)
No	1087	(96)
Tobacco user *	26	(2.3)
Yes	175	(15.5)
No	957	(84.5)
**Presenting Symptoms**		
Cough with other symptoms	101	(8.9)
Only cough	687	(60.7)
No cough but other symptoms	344	(30.4)
**Previous history of TB**		
Yes	84	(7.4)
No	1048	(92.6)
**History of TB of other family ^#^**		
Yes	118	(10.4)
No	1014	(89.6)
**Diabetes**		
Yes	244	(21.5)
No/Unknown ^$^	888	(78.5)
**HIV**		
Yes	13	(1.2)
No/Unknown ^$^	1119	(98.8)

**Abbreviation:** TB—Tuberculosis; PTBP—Presumptive TB patient; ACF—Active Case Finding, Diabetes = Diabetes Mellitus, HIV = Human Immunodeficiency Virus, * use in the last month, History of TB among any other household member within the last two years, ^$^ This group contains those tested without disease (No) and also those who were not tested for the disease (Unknown).

**Table 2:** Association of sociodemographic and clinical characteristics with not getting the TB diagnostic test (Sputum microscopy, CXR, CBNAAT) among the PTBPs identified by ACF activity in Kolkata Municipal Corporation, West Bengal, India during July–December 2018, n = 1132.

Variable	Total	Not Visiting the Health Facility, n (%) *	Unadjusted RR (95% CI)	Adjusted RR (95% CI) ^$^
**Total**	**1132**	**419 (37.0)**		
**Age in Years**				
0–14	36	10 (27.8)	1	1
15–29	206	63 (30.6)	1.1 (0.6–1.9)	1.3 (0.7–2.2)
30–44	323	117 (36.2)	1.3 (0.8–2.3)	1.5 (0.8–2.5)
45–59	280	93 (33.2)	1.2 (0.7–2.1)	1.4 (0.8–2.3)
60–74	184	64 (34.8)	1.3 (0.7–2.2)	1.4 (0.8–2.5)
75 and above	32	4 (12.5)	0.5 (0.2–1.3)	0.5 (0.2–1.5)
Not recorded	71	68 (95.8)	3.4 (2.0–5.9)	3.3 (1.9–5.6)
**Gender**				
Male	647	211 (32.6)	1	1
Female	485	208 (42.9)	1.3 (1.1–1.5)	1.3 (1.2–1.6)
**Alcohol use**				
Yes	45	28 (62.2)	1.7 (1.4–2.2)	1.7 (1.3–2.2)
No	1087	391 (36.0)	1	1
**Tobacco use**				
Yes	175	74 (42.3)	1.2 (1.0–1.4)	1.2 (1.0–1.4)
No	957	345 (36.1)	1	1
**Presenting Symptom**				
Cough with other symptoms	101	26 (25.7)	1.1 (0.7–1.6)	1.1 (0.8–1.6)
Only cough	687	312 (45.4)	1.9 (1.6–2.4)	1.8 (1.5–2.2)
No cough but other symptoms	344	81 (23.5)	1	1
**Previous history of TB**				
Yes	84	27 (32.1)	1	1
No	1048	392 (37.4)	1.2 (0.8–1.6)	1.0 (0.8–1.4)
**Family history of TB**				
Yes	118	70 (59.3)	1.7 (1.5–2.0)	1.5 (1.3–1.8)
No	1014	349 (34.4)	1	1
**Diabetes**				
Yes	244	63 (25.8)	1	1
No/Unknown ^#^	888	356 (40.1)	1.6 (1.2–1.9)	1.3 (1.0–1.6)
**HIV**				
Yes	13	1 (7.7)	1	1
No/Unknown ^#^	1119	418 (37.4)	4.9 (0.7–32.0)	6.0 (0.9–39.8)

* Row percentage, ^#^ This group contains those who were tested without disease (No) and also those who were not tested for the disease (Unknown), ^$^ cluster adjusted (wards) generalized linear (Poisson) model. **Abbreviation:** TB—Tuberculosis; PTBP—Presumptive TB patient; RR—Relative Risk; aRR—Adjusted Relative Risk; CI—Confidence Interval; ACF—Active Case Finding.

**Table 3:** Association of sociodemographic and clinical characteristics with getting positive TB diagnosis among those who underwent TB testing during the ACF activity in Kolkata Municipal Corporation, West Bengal, India during July–December 2018, n = 713.

Variable	Total	Diagnosed as TB, n (%) *	Unadjusted RR (95% CI)	Adjusted RR (95% CI) ^$^
**Total**	**713**	**177 (24.8)**		
**Age in years**				
0–14	26	10 (38.5)	2.7 (1.5–4.8)	2.2 (1.2–3.9)
15–29	143	70 (49.0)	3.4 (2.3–5.0)	2.4 (1.6–3.5)
30–44	206	47 (22.8)	1.6 (1.0–2.4)	1.3 (0.9–2.0)
45–59	187	27 (14.4)	1	1
60–74	120	19 (15.8)	1.1 (0.6–1.9)	1.0 (0.6–1.6)
75 and above	28	3 (10.7)	0.7 (0.2–2.3)	0.7 (0.2–1.8)
Not recorded	3	1 (33.3)	2.3 (0.4–11.9)	2.4 (0.4–14.2)
**Gender**				
Male	436	95 (21.8)	1	1
Female	277	82 (29.6)	1.4 (1.1–1.8)	1.1 (0.9–1.4)
**Alcohol user**				
Yes	17	4 (23.5)	1	1
No	696	173 (24.9)	1.1 (0.4–2.5)	1.3 (0.7–2.5)
**Tobacco user**				
Yes	101	9 (8.9)	1	1
No	612	168 (27.5)	3.1 (1.6–5.8)	2.2 (1.1–4.2)
**Presenting Symptoms**				
Cough with other symptoms	75	50 (66.7)	4.4 (3.3–5.8)	3.3 (2.4–4.4)
Only cough	375	57 (15.2)	1	1
No cough but other symptoms	263	70 (26.6)	1.8 (1.3–2.4)	1.4 (1.1–2.0)
**Previous history of TB**				
Yes	57	7 (12.3)	1	1
No	656	170 (25.9)	2.1 (1.0–4.3)	3.6 (1.7–7.6)
**Family history of TB**				
Yes	48	29 (60.4)	2.7 (2.1–3.6)	2.7 (2.0–3.6)
No	665	148 (22.3)	1	1
**Diabetes**				
Yes	181	54 (29.8)	1.3 (1.0–1.7)	1.2 (0.9–1.5)
No	532	123 (23.1)	1	1
**HIV**				
Yes	12	1 (8.3)	1	0.8 (0.1–5.8)
No/Unknown	701	176 (25.1)	3.0 (0.5–19.8)	1

* Row percentage, # This group contains those tested without disease (No) and also those who were not tested for the disease (Unknown), ^$^ cluster adjusted (wards) generalized linear (Poisson) model. **Abbreviation:** TB—Tuberculosis; RR—Relative Risk; aRR—Adjusted Relative Risk; CI—Confidence Interval; ACF—Active Case Finding; r = row percentage.
